# Supplementary material for: High Propagule Pressure and Patchy Biotic Resistance Control the Local Invasion Process of the Tree Ligustrum lucidum in a Subtropical Forest of Uruguay
Source: Plants (Basel). 2025 Mar 11;14(6):873. doi: 10.3390/plants14060873 (PMC11946171; doi:10.3390/plants14060873)
Supplement: Supplementary file 1 [file plants-14-00873-s001.zip › plants-3445564-supplementary.pdf]

Supplementary material

Table S1. Effects of past control activity on *L. lucidum* trees (fell 10 years ago) on its current recruitment. Best GLM models of density of *L. lucidum* seedlings, saplings, and poles in Ligustrum stands (invaded no controlled stands) and stands where adults of *L. lucidum* were controlled 2 (C2) and 10 (C10) years before our sampling. Distance from mother trees was also included in the models. The coefficient estimates for stand types were assessed with respect to C10 stands. Significance codes: '***' 0.001, '**' 0.01, '*' 0.05, '. 0.1, ' ' 1.

SEEDLINGS MODEL: glm(formula = Seedling ~ Distance + Stand + Distance:Stand, family = Poisson, data = datos)

	Estimate	Std. Error	z value	Pr(>|z|)	
(Intercept)	5.60474	0.03749	149.491	<2e-16	***
Distance	0.01134	0.01010	1.122	0.262	
StandC2	2.15236	0.06355	33.869	<2e-16	***
StandL	2.50795	0.03877	64.691	<2e-16	***
Distance:StandC2	-0.33535	0.01684	-19.913	<2e-16	***
Distance:StandL	-0.61055	0.01308	-46.694	<2e-16	***

Null deviance: 42293 on 25 degrees of freedom
Residual deviance: 21527 on 20 degrees of freedom
AIC: 21718 Best model

SAPLINGS MODEL: glm(formula = Saplings ~ Distance + Stand + Distance:Stand, family = Poisson, data = datos)

	Estimate	Std. Error	z value	Pr(>|z|)	
(Intercept)	2.07030	0.19948	10.378	< 2e-16	***
Distance	0.13153	0.04776	2.754	0.005883	**
StandC2	0.86220	0.24438	3.528	0.000418	***
StandL	3.26271	0.20305	16.068	< 2e-16	***
Distance:StandC2	0.39825	0.05374	7.411	1.25e-13	***
Distance:StandL	-0.59157	0.05598	-10.568	< 2e-16	***

Null deviance: 4472.4 on 25 degrees of freedom
Residual deviance: 1508.8 on 20 degrees of freedom
AIC: 1649.6 Best model

POLES MODEL: glm(formula = Poles ~ Distance + Stand + Distance:Stand, family = Poisson, data = datos)

	Estimate	Std. Error	z value	Pr(>|z|)	
(Intercept)	3.48066	0.09342	37.260	< 2e-16	***
Distance	0.19233	0.02120	9.071	< 2e-16	***
StandC2	-0.76954	0.22100	-3.482	0.000498	***
StandL	-1.04767	0.14844	-7.058	1.69e-12	***
Distance:StandC2	0.17778	0.04213	4.219	2.45e-05	***
Distance:StandL	-0.33762	0.05752	-5.869	4.38e-09	***

Null deviance: 1422.68 on 25 degrees of freedom
Residual deviance: 424.72 on 20 degrees of freedom
AIC: 543.66 best model
